# Bilateral Renal Oncocytoma: Active Surveillance Versus Partial Nephrectomy

**DOI:** 10.7759/cureus.22303

**Published:** 2022-02-16

**Authors:** Abha Sathe, David Lindars, Pranav Sathe, Rithvik Nallapareddy, Tim Grennan

**Affiliations:** 1 Urology, California Northstate University College of Medicine, Elk Grove, USA; 2 Hematology and Medical Oncology, California Northstate University College of Medicine, Elk Grove, USA

**Keywords:** active surveillance, core renal biopsy, partial nephrectomy, renal cell carcinoma, birt-hogg-dube syndrome, bilateral renal oncocytoma

## Abstract

Renal oncocytomas (ROs) are benign tumors comprising 16% of renal masses. Due to the overlapping phenotypes seen in RO and chromophobe renal cell carcinoma (RCC) and lack of specific clinical and laboratory characteristics of RO, physicians face a challenge when arriving at a definitive diagnosis of RO. ROs additionally appear indistinct from RCCs on CT scan, contributing further to the difficulty of arriving at a clear diagnosis of RO.

This is a case report of a 66-year-old man who presented with flank pain found to be related to bilateral ROs and underwent bilateral partial nephrectomies.

ROs are benign small renal masses that often pose a diagnostic challenge since preoperative diagnosis can be difficult to achieve. Given advancements in technology, active surveillance with core renal biopsy is a promising approach to accurately diagnose and manage ROs conservatively. The application of these techniques has wide-reaching implications for patients and physicians by reducing the need for a potentially harmful surgery and creating a cost-effective way to manage a diagnosis.

## Introduction

Renal oncocytoma (RO) is a highly differentiated tumor that originates from the distal renal tubule and accounts for 4.3% of solid renal masses and 16% of all renal masses [1,2]. While ROs usually occur unilaterally, 4-5% of ROs occur bilaterally [2]. With few cases of documented metastasis, ROs have a benign clinical course with excellent long-term outcomes and 100% disease-specific survival [3]. On histology, ROs present as uniform, round, or polygonal tumor cells that exhibit a granular eosinophilic cytoplasm [4].

A renal mass is a nonspecific finding on computed tomography (CT) requiring investigation to distinguish RO from renal cell carcinoma (RCC) and chromophobe renal cell carcinoma (chRCC). It is reported that up to 30% of the small renal masses (SRMs) detected on routine imaging are benign, but most are still treated without a tissue diagnosis. “Segmental enhancement inversion” has shown specificity in distinguishing between RCC and oncocytoma in a size-dependent manner [5]. The diagnosis of RO is complicated by the possibility of hybrid tumors consisting of both RCC and RO features, which can be found in up to 32% of the patients [6]. Patients with hybrid tumors often present with constitutional symptoms and gross hematuria when compared to patients with only RO, without coexisting RCC [7].

There are wide implications for a patient who might elect to undergo aggressive therapy given the possibility of a renal malignancy. Partial nephrectomy (PN) is suggested for patients with a renal tumor <4 cm in an attempt to preserve as much renal parenchyma as possible, while radical nephrectomy is recommended for tumors larger than 4 cm [3]. PN is often performed for ROs for histological diagnosis and removal of the dominant mass. The patient is subsequently followed post-partial nephrectomy with active surveillance (AS), which includes routine imaging tailored to the patient’s profile and risk factors to monitor tumor growth after initial complete staging, even in the presence of diffuse renal lesions [8].

If multiple masses are detected in the same kidney, clinicians often perform enucleation of all tumors due to concerns about underlying malignancy despite data showing that few are associated with malignant behavior [9,10]. Given the evolving urological field and technology, however, there has been a push toward AS and biopsy to avoid over-treatment of the patient. Renal core biopsies had an accuracy of 97.1% in identifying malignancies in a study of 442 biopsies. Since urologists can perform renal core biopsies, the number of healthcare employees a patient must encounter to receive appropriate care is minimized. The complications of the biopsy are minor including hematomas detected by ultrasound, reduction in hemoglobin concentrations, macrohematuria, or major ones where patients will require blood transfusions, arterial-venous fistulas, or angiographic intervention [11].

Due to the overlapping phenotypes seen in RO and chRCC and lack of specific clinical and laboratory characteristics of RO, physicians face a challenge when arriving at a definitive diagnosis of RO [2]. ROs additionally appear indistinct from RCCs on CT scan, contributing further to the difficulty of arriving at a clear diagnosis of RO. A barrier to the diagnosis and management of ROs is metastasis further complicating the treatment options available to patients with recently diagnosed renal masses. Renal metastasis can be classified as synchronous or metachronous metastatic disease. Synchronous metastatic disease involves the presence of primary tumor and metastasis at the time of diagnosis, whereas metachronous involves the development of metastatic disease after surgery.

In this article, we present a case of bilateral ROs in which the patient underwent bilateral PNs instead of conservative management due to the inability to clearly diagnose RO. While multicentricity, increased incidence, and asymptomatic course may complicate the diagnosis of RO, the condition is benign and non-emergent. Treatment should prioritize conservative approaches above other invasive options to optimize patient comfort and safety.

## Case presentation

A 66-year-old man presented to the emergency department with right flank and lower back pain, which worsened with movement/position changes and was not relieved with ibuprofen use. He denied hematuria, dysuria, abdominal pain, nausea or vomiting, and trauma. His medical history was significant for hypertension, hyperlipidemia, actinic keratosis, hypothyroidism, allergic rhinitis, acquired spondylolisthesis, obesity, and chronic gout with tophi. There was no clinical evidence of tuberous sclerosis and Birt-Hogg-Dube (BHD) syndrome. He was a lifelong nonsmoker and consumed 10 drinks of alcohol per week. The patient’s vital signs and physical examination were unremarkable except for a blood pressure of 165/95 mm Hg and pulse of 95 bpm. Complete blood count and comprehensive metabolic panel were unremarkable except for elevated blood urea nitrogen of 22. Urinalysis showed presence of trace ketones and high protein content.

**Figure 1:**
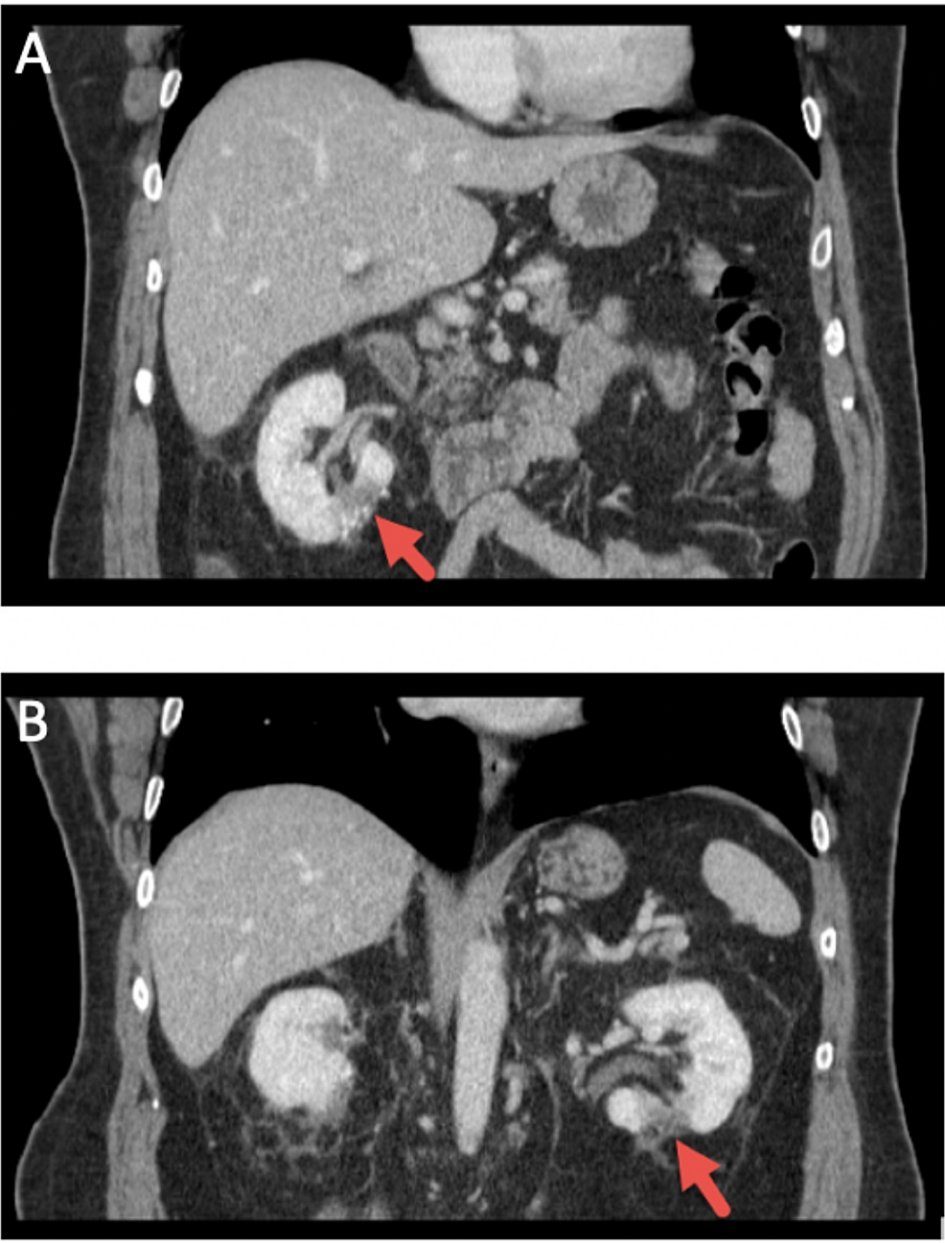
CT abdomen pelvis of a 66-year-old man with bilateral renal oncocytoma. (A) Arrow points toward a 3.0 cm mass in the lower pole of the right kidney on CT. (B) Arrow points toward a 3.3 cm isodense mass on the left lower kidney on CT. CT, computed tomography.

CT abdomen pelvis study showed a 3.0 cm mass in the lower pole of the right kidney and a 2 cm cyst on the right kidney (Figure 1A) as well as a 3.3 cm isodense mass in the lower pole of the left kidney (Figure 1B). For his left renal mass, the patient underwent successful robotic PN. Surgical pathology report of the resected left renal mass revealed the presence of RO. 

**Figure 2:**
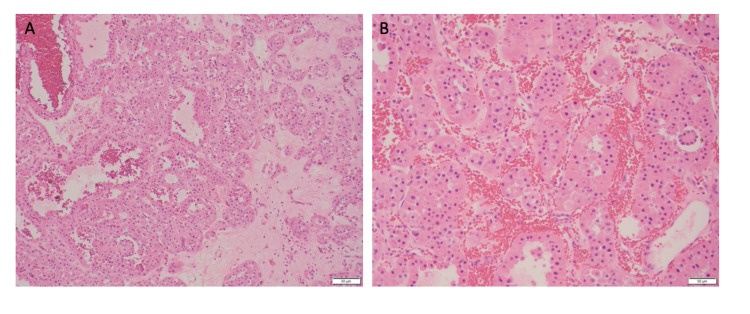
Histology of the resected right lower pole mass indicating renal oncocytoma. (A) Microscopic (×100) analysis of H&E-stained tumor shows typically small solid nests or tubules in a loose connective tissue stroma. The tumor cells demonstrate dense granular cytoplasm with round and regular nuclei. (B) Microscopic (×200) analysis of H&E-stained tumor shows typically small solid nests or tubules in a loose connective tissue stroma. The tumor cells demonstrate dense granular cytoplasm with round and regular nuclei. H&E, hematoxylin and eosin.

In light of the diagnosis of a left RO, the right renal mass was provided with a 75% likelihood of RO. The patient was counseled on the option to pursue PN or biopsy followed by observation. Despite the overwhelming likelihood of RO, the patient opted for a robotic PN to remove the right renal mass and ensure removal of any possible malignancy. Expectedly, the surgical pathology report of the resected right renal mass confirmed the presence of an RO, showing similar morphology as the resected left mass (Figure 2A, 2B). Postoperative course for both surgeries was uneventful and the patient was discharged within one day of the surgery. He has continued with observation without concern for recurrence. 

With a diagnosis of bilateral ROs, family history of melanoma and prostate cancer, and medical history of actinic keratoses and multiple nonmelanoma skin cancer, the patient sought cancer genetic counseling to evaluate a possible familial disposition to cancer due to BHD syndrome. Our patient tested negative for the FLCN gene, which is found in 84% of BHD-diagnosed patients.

## Discussion

Our patient elected to undergo surgery to remove the contralateral right renal mass after a histologic diagnosis of RO for the left renal mass. In patients who have undergone PN with a diagnosis of RO, there is an increased likelihood of benign neoplasm and decreased risk of RCC, similar to that of the general population [12]. After our patient’s left PN, given the diagnosis of RO and the absence of constitutional symptoms or gross hematuria, the chance of RCC or hybrid tumor with RO was decreased. Because the left renal mass was 3.3 cm in size and undiagnosed prior to intervention, the mass was more concerning for RCC; however, with the diagnosis of RO in the left kidney and the decreased size of the right renal mass at 2 cm, there was a negligible risk of metastasis, which warranted AS rather than intervention. With the initial diagnosis of left RO, Childs et al. would not recommend intervention.

The patient could have undergone a confirmatory biopsy of the contralateral right renal mass with AS imaging protocols instead of undergoing a right PN [13]. PNs are associated with complications such as postoperative hemorrhage and urine leak, such that for every 1 cm increase in tumor diameter, the risk of postoperative hemorrhage increases by 45% [14]. AS by sonogram or core needle biopsy has shown to be a safe alternative to definitive treatment in terms of ROs with 70% diagnostic accuracy [15]. One study reported the risk of metastatic disease for patients with renal tumors <3 cm to be negligible and concluded that tumor size was significantly associated with synchronous and metachronous metastases following nephrectomy [16]. Incidence of oncocytoma increases to 18% when tumors are less than 4 cm in size [17]. Therefore, patients with SRMs with low risk of metastasis and comorbidities can be followed with imaging until the tumor grows or reaches a size of >2.5 cm [16]. ROs have a benign course with a growth of 0.20 cm or less per year. When followed with AS, ROs are seen without metastasis for at least 18 years and up to at least 33 years after a simple excision [18]. Previous reports of metastasized oncocytomas are now being re-classified as potentially RCC or chRCC [19]. Overall, the excellent prognosis of oncocytomas supports the idea that AS with biopsy is an appropriate approach for bilateral RO after histological confirmation of one RO with PN in cases of bilateral ROs. 

## Conclusions

This case report presents a patient with bilateral ROs who underwent bilateral PNs with histological diagnosis after each nephrectomy. After left PN, the patient's right RO could have been managed with AS and renal core biopsy, as this method has been shown to be a safe, cost-effective, and least invasive option in select patients. Further studies are needed to determine the long-term consequences of AS in those without limited life expectancy and few to no comorbidities.
